# Electrolyte homeostasis in pregnancy: from physiological adaptations to clinical disturbances — a nephrologist’s perspective

**DOI:** 10.3389/fneph.2026.1773415

**Published:** 2026-03-26

**Authors:** Priti Meena, Aisha Batool

**Affiliations:** 1 Department of Nephrology, All India Institute Medical Sciences, Bhubaneswar, India; 2 Division of Nephrology, Medical College of Wisconsin, Hub for Collaborative Sciences, Milwaukee, WI, United States

**Keywords:** AVP, electrolytes, osmoregulation, pregnancy, sodium

## Abstract

Electrolyte homeostasis in pregnancy undergoes several important remodellings driven by systemic vasodilation, activation of neurohormonal pathways, increased glomerular filtration, altered tubular transport, and active maternal–fetal mineral exchange. These coordinated adaptations enable plasma volume expansion, mainta*in utero*placental perfusion, and support fetal growth, yet they narrow compensatory reserves and shift normal biochemical reference thresholds. As a result, reliance on non-pregnant laboratory norms can misclassify abnormalities, delaying recognition of clinically important disturbances. Understanding pregnancy-specific physiology is therefore essential for accurate diagnosis, monitoring, and therapeutic decision-making. This review provides an integrated nephrology-focused synthesis of normal adaptive mechanisms and disorder-specific pathophysiology across sodium–water, potassium, magnesium, and calcium balance. We summarize expected gestational changes, including the reset osmostat and AVP-mediated free-water retention causing a physiological fall in serum sodium, changes in potassium homeostasis and magnesium homeostasis, and the doubling of intestinal calcium absorption driven by increased calcitriol to meet third-trimester skeletal mineralization. We further review common clinical disorders of water and sodium, potassium, calcium, and magnesium. The review provides a comprehensive pregnancy-specific interpretation of electrolyte values, diagnostic evaluation strategies, and targeted management tailored to maternal and fetal safety aimed at improving clinical vigilance and optimizing outcomes.

## Introduction

Pregnancy induces significant systemic, renal, and endocrine adaptations that support fetal growth while maintaining maternal homeostasis. These changes cause alterations in electrolyte balance, fluid distribution, and acid–base physiology (1). Conventional laboratory reference ranges derived from non-pregnant healthy individuals may therefore misclassify normal gestational changes as abnormal or sometimes even overlook true pathology (2, 3). For example, mild reductions in serum sodium and creatinine may be physiological in pregnancy, whereas values considered “normal” outside pregnancy may signal disease. Because early symptoms of electrolyte disturbances are often nonspecific and easily attributed to typical gestational discomfort, delayed recognition can result in serious maternal and fetal complications, including seizures, arrhythmias, growth restriction, and neurologic injury (4). A clear understanding of pregnancy-specific physiology is therefore essential for accurate diagnosis and appropriate management. This review provides a nephrology-centred synthesis of sodium–water, potassium, magnesium, calcium and phosphorus disorders in pregnancy, integrating physiological adaptation with practical diagnostic and therapeutic implications, and emphasizing the need for pregnancy-specific laboratory interpretation and enhanced clinical vigilance.

## Osmoregulation and sodium–water homeostasis in pregnancy

Normal pregnancy is characterized by significant systemic and renal hemodynamic adaptations that facilitate progressive sodium retention and plasma volume expansion while maintaining normal fetal perfusion. These changes are reflections of a unique physiological state in which systemic vasodilation, reduced total peripheral vascular resistance, and arterial underfilling coexist with marked fluid accumulation and declining blood pressure conditions that is considered pathologic in states other than pregnancy (5). The concept of *effective arterial blood volume* (EABV) is central to explaining these adaptations; renal sodium and water handling is regulated primarily by perceived, rather than absolute, blood volume. Vasodilation and reduced EABV activate the sympathetic nervous system, the renin–angiotensin–aldosterone system (RAAS), and non-osmotic release of arginine vasopressin (AVP), thereby enhancing renal sodium and water retention to restore perfusion pressure (6).

In early gestation, systemic vasodilation is primarily driven by nitric oxide, relaxin, and hormonal modulation results in decreased vascular resistance and arterial underfilling, which in turn results in sodium retention and plasma volume expansion. Elevated levels of estrogen and progesterone are strongly associated with systemic vasodilation, and concentrations of both hormones rise substantially during pregnancy. Relaxin, a peptide hormone produced by the corpus luteum, peaks toward the end of the first trimester and subsequently decline to a stable intermediate concentration for the remainder of pregnancy. Relaxin has been shown to exert endothelium-dependent vasodilatory effects, particularly on small resistance arteries, and is an important mediator of the early gestational vasodilatory response. Glomerular filtration rate (GFR) rises by nearly 50% by the end of the first trimester, increasing the filtered sodium load by 20,000–30,000 mmol/day (7). Despite this increase in filtered sodium, absolute and fractional excretion decrease due to enhanced tubular sodium reabsorption via aldosterone-mediated activation of epithelial sodium channels (ENaC), increased deoxycorticosterone synthesis, and angiotensin II–dependent transporter up-regulation. The net result is cumulative retention of approximately 900–1,000 mmol of sodium and 8–10 L of water over the course of pregnancy (8).

**Figure 1:**
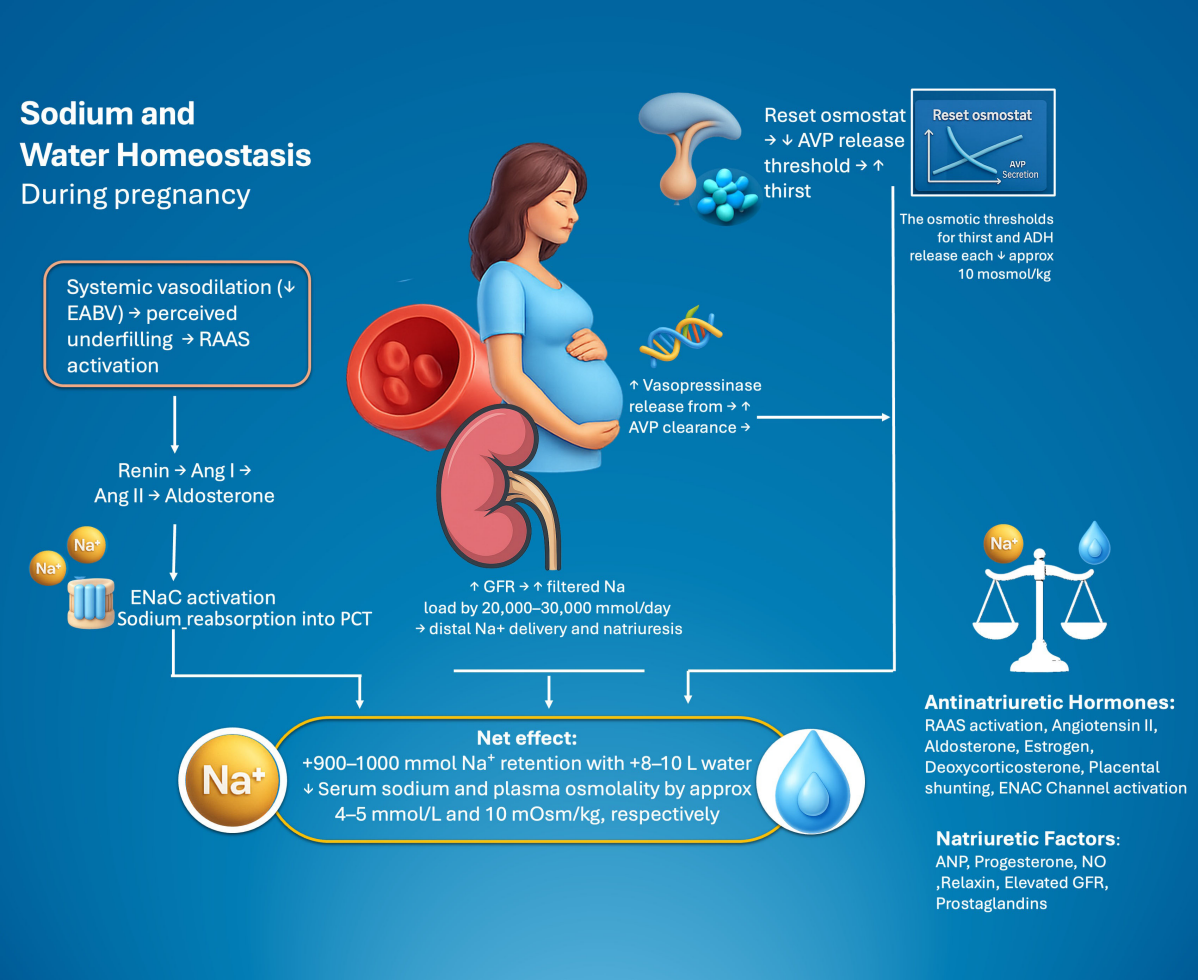
Osmoregulation and sodium balance during pregnancy. EABVm, Effective Arterial Blood Volume; RAAS, Renin–Angiotensin–Aldosterone System; Ang I, Angiotensin I, Ang II, Angiotensin II; AVP, Arginine Vasopressin; GFR, Glomerular Filtration Rate; ENaC, Epithelial Sodium Channel; NO, Nitric Oxide; ANP, Atrial Natriuretic Peptide; DOC, Deoxycorticosterone; ENaC, Epithelial Sodium Channel.

On background of these physiological changing developing in pregnancy, a reduction in serum sodium and plasma osmolality by approximately 4–5 mmol/L and 10 mOsm/kg, respectively is observed during pregnancy (5–7). These changes are driven by a reset osmostat phenomenon whereby osmotic thresholds for thirst and AVP release shift downward. The presence of a functional fetoplacental unit is essential to develop such physiological changes, as simulated pregnancy without the placenta fails to induce this osmostatic resetting (9). β-hCG and relaxin are implicated mechanistically, contributing to AVP sensitivity modulation and thirst threshold reduction (9, 10). Simultaneously, plasma vasopressinase, an enzyme secreted by the placenta increases three- to fourfold by mid-gestation and metabolizes AVP, partially explaining why AVP concentrations remain unsuppressed despite hypo-osmolality and why osmotic AVP responses are blunted late in pregnancy (10). **Figure 1** summarizes the osmoregulation and sodium homeostais during pregnancy.

Water retention continues until plasma osmolality falls below the newly established thirst threshold, generating a new steady state. Although total plasma volume increases by 50–70%, pregnant women demonstrate uncertainty in volume sensing, perceiving themselves as normovolemic despite dramatic blood volume rise this observation that challenges classical understanding of volume-mediated sodium regulation (11, 12). Peripheral edema should therefore not be interpreted as a reliable indicator of intravascular volume expansion. In women with CKD, baseline hematocrit and serum albumin monitoring in early gestation is useful, as increases indicate volume contraction. Clinical context and details of sodium disorders (13–18) are shown in **Table 1**..

**Table 1:** Hyponatremia and hypernatremia in pregnancy.

Feature	Hyponatremia	Hypernatremia
Physiologic Reference Range	130–140 mmol/L (lower than non-pregnant 135–145 mmol/L)	No physiologic upward shift
Definition	<130 mmol/L	>145 mmol/L
Primary Mechanism	Dilutional (impaired free-water excretion, AVP activity, osmostat resetting), antidiuretic effects of oxytocin, particularly in late pregnancy and labour	Mostly due to severe volume depletion due to gastrointestinal losses, uncontrolled diabetes insipidus or insufficient fluid intake
Pregnancy-Specific Drivers	Persistent AVP activity (inability to excrete excess free water), increases AVP release via pain, stress, and nausea during labour, oxytocin administration (AVP-like effect), excessive hypotonic IV fluids during labour	Gestational diabetes insipidus is characterized by polyuria and polydipsia due to impaired ADH activity or renal insensitivity to ADH. During pregnancy, placental trophoblast mass increases nearly 1000-fold, resulting in markedly increased production of vasopressinase. Vasopressinase activity rises 40–50-fold, and although hepatic degradation capacity also increases approximately four-fold, any imbalance between production and clearance precipitates excessive ADH destruction and free-water loss. Compensatory mechanisms, including increased posterior pituitary ADH secretion and up-regulation of renal aquaporin-2, are frequently insufficient to maintain water homeostasis.
High-Risk Settings	Prolonged labour; High-dose oxytocin; Hypotonic fluids; Preeclampsia/HELLP	Third trimester; Multiple gestation; Acute fatty liver; Preeclampsia/HELLP
Maternal Clinical Features	Clinical manifestations may range from mild nonspecific symptoms nausea, headache, apathy, lethargy to advanced neurologic compromise including agitation, seizures, respiratory arrest, and coma.	Polyuria, polydipsia, dehydration, altered sensorium
Fetal Implications	Maternal hyponatremia rapidly equilibrates across the placenta, causing fetal hyponatremia and risk of neonatalencephalopathy.	Risk of fetal dehydration if severe/prolonged
Diagnostic Approach	Plasma and urine osmolality, Urine sodium; Assess acuity; Exclude hyperglycemia	Confirm plasma osmolality, Urine osmolality,
Precautions	Isotonic rather than hypotonic IV solutions should be used where possible; avoiding dextrose 5% as an oxytocin carrier; restricting total fluid input in labouring women (e.g. <2–2.5 L over labour unless there is a clear indication); and routine fluid balance monitoring with serum sodium measurements in prolonged labour, high-dose oxytocin use,	Conditions such as multiple gestation, acute fatty liver of pregnancy, preeclampsia/HELLP, and liver dysfunction potentiate GDI by reducing vasopressinase clearance, while central ADH deficiency (e.g., prolactinoma, craniopharyngioma, Sheehan syndrome) or nephrogenic defects may unmask symptoms.
Management Principles	Same principles as in non-pregnant individuals, Acute: hypertonic saline; Avoid hypotonic fluids; Restrict labour fluids; Monitor sodium	Restore volume, Desmopressin (DDAVP) in DI is resistant to vasopressinase degradation, is safe in pregnancy and breastfeeding and is used with careful monitoring to avoid iatrogenic hyponatremia.

## Potassium homeostasis in pregnancy

Pregnancy induced physiological increase in GFR, substantially increases the filtered load of electrolytes, including potassium. Despite this rise in filtered potassium load and a marked increase in plasma aldosterone concentrations, both absolute and fractional urinary potassium excretion remain remarkably constant across trimesters maintain the gestational potassium homeostasis (19). This is supported by contemporary cohort data showing minimal variation in serum potassium between trimesters, with values ranging narrowly from approximately 3.67 to 3.82 mmol/L (20). The apparent paradox of markedly elevated aldosterone concentrations without progressive kaliuresis reflects the balance between forces that promote potassium secretion and adaptive mechanisms that restrain it. Increased GFR augments distal tubular flow and sodium delivery to distil nephron. In isolation, this would be expected to enhance lumen negativity, stimulate ROMK and BK channel activity, and increase urinary potassium losses. However, pregnancy is not a state of isolated mineralocorticoid action. The simultaneous and substantial rise in progesterone competitively antagonizes the mineralocorticoid receptor, attenuating aldosterone-dependent ENaC activation and thereby limiting the electrogenic gradient that drives potassium secretion. The net effect is preservation of serum potassium despite high circulating aldosterone and expanded extracellular volume (19). The net effect is that serum potassium levels remain stable through adaptive modulation of distal potassium reabsorption and secretion (21).

## Hypokalemia in pregnancy

Although hypokalemia is uncommon in healthy pregnancies, it can occur in association with intercurrent illness, increased gastrointestinal losses, or shifts in transcellular potassium distribution. Large obstetric cohorts estimate the incidence of hypokalemia during pregnancy or the immediate postpartum period to be approximately 0.36 percent of total births (22, 23). In a study of 110 affected women, 91 % had mild to moderate hypokalemia (2.6–3.1 mmol/L), while 9 % had severe hypokalemia (<2.6 mmol/L) (23). Hyperemesis gravidarum, vomiting, hypertensive disorders of pregnancy, and postpartum hemorrhage were the leading associations (22). In a study of Diabetic ketoacidosis in pregnancy more than half of the episodes of DKA had hypokalemia (24). Women with underlying renal tubular disorders, such as Bartter or Gitelman syndromes, may experience exacerbation of hypokalemia. In such cases, hypokalemia may be severe and associated with metabolic alkalosis, necessitating careful maternal–fetal monitoring and aggressive electrolyte repletion (25). Clinically, the sign and syptoms of hypokalemia in pregnancy mirror those in nonpregnant adults, including cardiac arrhythmias, muscle weakness. Treatment includes addressing underlying causes, oral potassium supplementation, and intravenous replacement for severe or symptomatic hypokalemia, with continuous maternal and fetal monitoring. In patients with hyperemesis gravidarum and starvation ketosis, careful supplementation of thiamine along with electrolytes and gradual increase in calorie intake should be followed given the risk of refeeding syndrome.

## Hyperkalemia in pregnancy

Occurrence of hyperkalemia in pregnancy signals towards impaired aldosterone production or action, reduced distal delivery, or pharmacologic blockade of the ENaC–aldosterone pathway. Heparin is a clinically important cause of hyperkalemia in obstetric practice. Both unfractionated heparin and low–molecular-weight heparins can suppress aldosterone synthesis, producing hyperkalemia within days to weeks of initiation (26). Diabetes mellitus and concomitant use of co-trimoxazole was associated with a higher incidence of hyperkalemia (27, 28). Type 4 renal tubular acidosis, although uncommon in pregnancy, may occur in women with diabetic nephropathy or adrenal insufficiency, presenting with mild hyperkalemia and non–anion-gap metabolic acidosis (29). Hyperkalemia can also be the complication of Acute kidney injury from preeclampsia, sepsis, or hemorrhage or any other etiology.

## Magnesium homeostasis in pregnancy

Pregnancy introduces complex physiological changes in magnesium handling, driven by plasma volume expansion, fetal-placental metabolic demands, and alterations in renal tubular reabsorption, all contributing to a characteristic slight fall in serum magnesium concentration (30, 31). Serum magnesium levels have been reported to decline progressively during gestation in some studies (32–34). In a study by Orlova S et al, including 983 pregnant women the prevalence of hypomagnesemia (magnesium serum level cut-off < 0.66 mmol/L/< 0.8 mmol/L) was 34.0%/78.9% (32). Another study showed median plasma Mg values of 0.69 mmol/L in mid-pregnancy (~17 weeks) and 0.63 mmol/L later (~26 weeks) in a large (n≈1,756) Chinese cohort suggesting the trend of decline (33). These reductions reflect several physiological adaptations. Hemodilution due to plasma volume expansion seems to be an important contributor, as shown in studies where the decline in serum proteins paralleled the decline in total serum magnesium (34). However, hemodilution alone cannot fully explain the observed changes (35). In a study by Handwerker SM et al, ionized magnesium (IMg²^+^) fell from 0.53 mmol/L in the first trimester to a nadir of 0.49 mmol/L in the third trimester, although IMg²^+^ remained tightly regulated at ~66% of total Mg, indicating selective mobilization of intracellular and bone stores to maintain physiological function (36). Renal handling of magnesium is also altered. Increased GFR during pregnancy increases the filtered load of magnesium, yet urinary magnesium excretion paradoxically remains stable across trimesters.

A uniquely pregnancy-specific mechanism is the maternal–fetal magnesium transfer. From mid-gestation onward, approximately 4.5 mg/day of magnesium is actively transported across the placenta (37). Fetal Mg concentrations exceed maternal levels, supporting an active transport mechanism, independent of protein binding differences.

## Magnesium disorders in pregnancy

Pregnant women are at specially at higher risk of developing magnesium deficiency. Hypomagnesemia during pregnancy is usually due to nutritional deficiency, gastrointestinal loss, or chronic maternal depletion rather than intrinsic renal magnesium wasting. Conditions such as hyperemesis gravidarum, poor oral intake, and diarrheal illnesses are common drivers, often accompanied by other electrolyte disturbances. Chronic primary magnesium deficiency is frequent in women, as studies had shown about 20% of women are receiving low intakes of magnesium and consuming less than two-thirds of the RDA (38). The clinical associations of hypomagnesemia mirror those in nonpregnant patients neuromuscular irritability, arrhythmias, and refractory hypokalemia but may have amplified consequences for maternal–fetal physiology. Maternal magnesium deficiency has been implicated as a potential contributor to hypertensive disorders of pregnancy, uterine hyperexcitability, preterm labor, impaired fetal growth, and gestational diabetes (28, 33, 38). The interplay of magnesium with vascular and uterine physiology also becomes clinically relevant. Magnesium modulates vasomotor tone by antagonizing calcium-dependent vasoconstriction, and decreased intracellular Mg may predispose to vasospasm and endothelial dysfunction (39). Treatment of hypomagnesemia in pregnancy should be focused on both correction and addressing the underlying cause. Oral magnesium salts are preferred for mild, asymptomatic cases. In symptomatic patients or severe deficiency intravenous magnesium sulfate is the treatment of choice. Serum magnesium should be monitored for toxicity specially in women with preeclampsia and renal impairment.

## Hypermagnesemia in pregnancy

Hypermagnesemia in pregnancy is almost exclusively iatrogenic, resulting from intravenous magnesium sulfate therapy used for preeclampsia, eclampsia prophylaxis, or tocolysis. Serum Mg >3.5 mmol/L impairs deep tendon reflexes; >5.0 mmol/L causes respiratory depression; extremely high levels may lead to conduction block, maternal cardiac arrest, and fetal hypotonia or CNS depression. In most cases the fetal increase is asymptomatic, though severe maternal toxicity may be mirrored in the neonate (40). Monitoring serum Mg correlates poorly with intracellular toxicity thresholds, emphasizing the importance of clinical neurologic assessment.

## Calcium and phosphorus homeostasis in pregnancy

Calcium and phosphorus homeostasis undergoes important physiological adaptation during pregnancy to support fetal skeletal development while maintaining maternal neuromuscular stability. By term, the fetal skeleton accrues approximately 28–30 g of calcium, with nearly 80% transferred during the third trimester, coinciding with rapid ossification of the collagen matrix (41). In mother several changes in intestinal absorption, renal handling, and skeletal turnover takes place to meet the demands without compromising maternal serum ionized calcium. Although total serum calcium declines due to hemodilution and hypoalbuminemia, albumin-corrected and ionized calcium concentrations remain essentially unchanged throughout pregnancy, suggesting that biochemical interpretation must rely on ionized or corrected values rather than total calcium (42).

## Endocrine regulation

**Figure 2:**
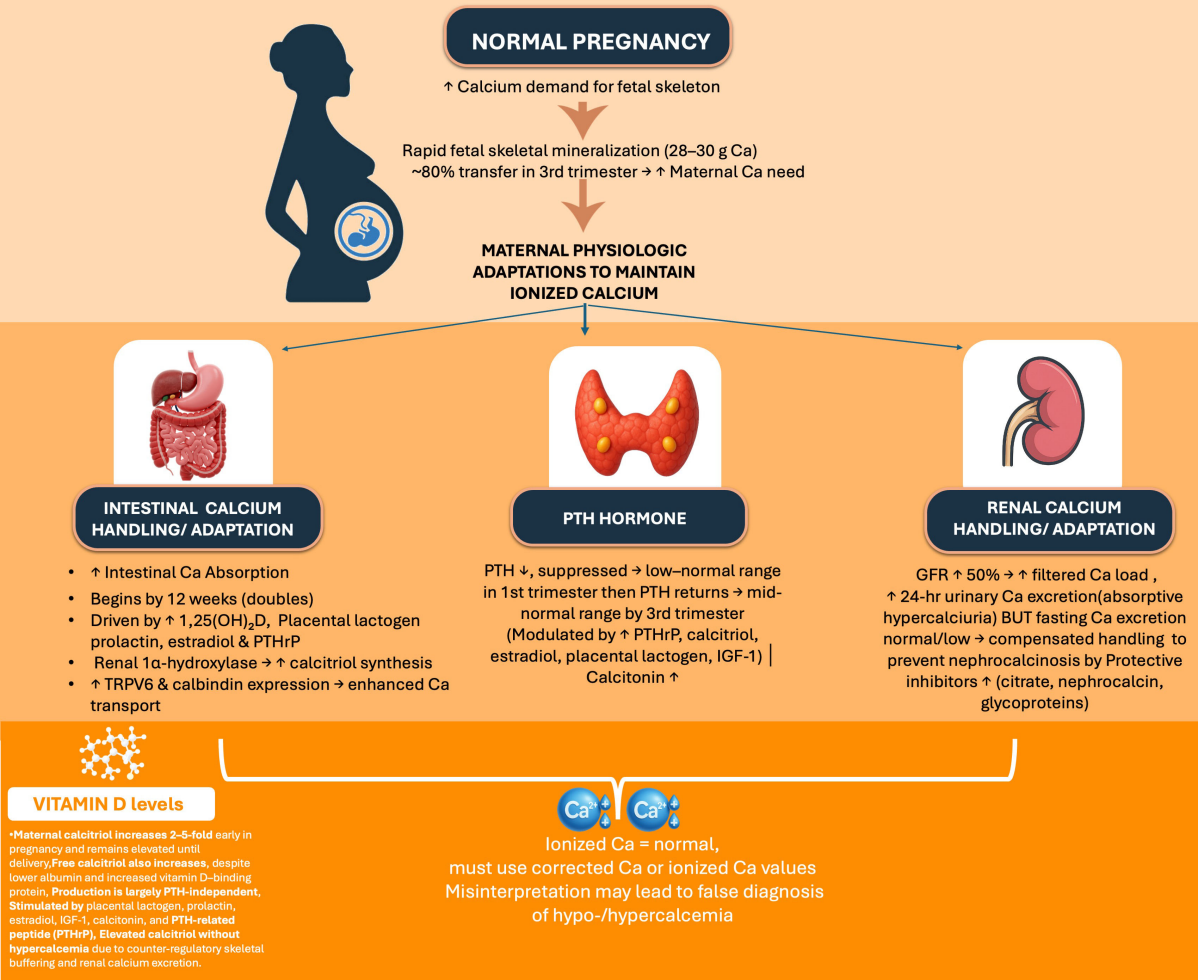
Calcium homeostasis during pregnancy. Ca, Calcium; TRPV6, Transient Receptor Potential Vanilloid Channel 6; 1,25(OH)_2_D, 1,25-Dihydroxyvitamin D (Calcitriol); 25(OH)D, 25-Hydroxyvitamin D; VDBP, Vitamin D Binding Protein; PTH, Parathyroid Hormone; PTHrP, Parathyroid Hormone–Related Peptide; IGF-1, Insulin-Like Growth Factor 1; PCT, Proximal Convoluted Tubule.

A key adaptation to maintain calcium homeostasis in pregnancy is marked increase in intestinal calcium absorption, which doubles by 12 weeks of gestation, preceding the peak fetal demand later in pregnancy. This increase correlates with a two- to five-fold rise in circulating 1,25-dihydroxyvitamin D (calcitriol) that begins in early gestation and persists until delivery (43). Calcitriol levels rise despite declining parathyroid hormone (PTH), indicating PTH-independent up-regulation of 1-α hydroxylase. Unlike the non-pregnant state, where PTH drives calcitriol production, intact PTH levels are suppressed into the low-normal range or even below normal during the first trimester, rising gradually to mid-normal levels by the third trimester (44). This endocrine reprogramming indicates pregnancy as a state of PTH-independent calcitriol synthesis. This regulation is mostly driven by hormonal influences from PTH-related peptide (PTHrP), placental lactogen, estradiol, prolactin, IGF-1, and FGF-23, which are probably together stimulating 1-α hydroxylase activity in the maternal kidney, placenta, and decidua. An increase of maternal Vitamin D binding protein (VDBP) concentrations is also observed (45, 46). (See **Figure 2**) Maternal 25-hydroxyvitamin D crosses the placenta and determines fetal vitamin D status, while placental expression of vitamin D receptor and metabolic enzymes demonstrates a critical immunomodulatory interface at the fetoplacental barrier (47).

Calcitonin levels rise significantly during pregnancy, produced from hypertrophied thyroid C-cells, breast tissue, and the placenta. In a cohort of 56 pregnant women, calcitonin was elevated in 72%, remaining high for 48 hours postpartum (48). Although proposed to protect maternal skeleton from excessive resorption, human studies have not confirmed a definitive protective skeletal action, as calcitonin-deficient mice maintain normal calcium metabolism during pregnancy. Increased intestinal absorption is accompanied by greater renal calcium excretion, an increase in 24-hour urinary calcium excretion is observed from early gestation, whereas fasting urine calcium remains normal or low, supporting the concept of absorptive hypercalciuria (49). Increased GFR augments filtered calcium load, and dissociation between sodium and calcium reabsorption in Henle’s loop further enhances calcium excretion. Despite urinary supersaturation, stone formation is rare due to increases in citrate, nephrocalcin, and acidic glycoproteins, potent inhibitors of calcium crystallization. However, these adaptations sometimes contribute to the higher incidence of nephrolithiasis in pregnancy in predisposed individuals.

Phosphate remains essential for fetal skeletal mineralization and cellular energy metabolism, and a physiological maternal–fetal gradient is maintained, with fetal phosphate concentrations approximately 0.5 mmol/L higher than maternal levels to facilitate skeletal accretion (50). In normal pregnancy, maternal serum phosphate typically remains within the non-pregnant reference range. Mild hypophosphatemia has been reported in a minority of women, with prevalences of approximately 3% in the first trimester and rising toward the mid-gestation period in some series (51). Amniotic fluid phosphate concentrations have been shown to decline with advancing gestation, likely due to progressive fetal uptake (51). Increased glomerular filtration may augment filtered phosphate load, and urinary phosphate excretion may rise in late pregnancy (52). In contrast, reduced phosphaturia and higher circulating phosphate levels have been described in severe preeclampsia, likely reflecting impaired renal function rather than primary disturbances in phosphate regulation (53). Abnormal phosphate values should therefore prompt evaluation for underlying renal dysfunction, hypertensive disorders, nutritional deficiency, or metabolic disease rather than being attributed to physiological gestational adaptation alone.Calcium disorders in pregnancy.

Hypocalcemia principally results from inadequate dietary calcium or vitamin D deficiency, rather than failure of physiologic adaptation. Persistent hypocalcemia stimulates fetal PTH, leading to subperiosteal bone resorption, intrauterine fractures, bowing of long bones, and low birth weight (54). Treatment requires aggressive replacement with calcium and vitamin D to ensure fetal skeletal integrity and prevent neonatal hypocalcemia. Hypercalcemia is rare but could be clinically significant, importantly when due to previously unrecognized primary hyperparathyroidism, excessive vitamin D intake, or granulomatous disease. It typically presents with nonspecific symptoms masking diagnosis nausea, vomiting, anorexia, and neuropsychiatric manifestations. Severe hypercalcemia may precipitate nephrolithiasis, pancreatitis, arrhythmias, pre-eclampsia, and fetal demise (55). Management centers on hydration and calcitonin, as bisphosphonates and denosumab are contraindicated.

**Table 2** shows comparison of important laboratory values during pregnancy and non-pregnant state.

**Table 2:** Comparison of important laboratory values during pregnancy and non-pregnant state.

Laboratory parameter	Non-pregnant state	During pregnancy
Arterial pH	7.40	7.44
Arterial PCO 2 (mm Hg)	40	30
Serum Bicarbonate (mmol/L)	24	20
Serum Sodium (mmol/L)	140	135
Serum osmolality (mOsm/kg)	285	275
Serum total protein (g/dl)	7.0	6.0
Serum Creatinine (mg/dl, µmol/l)	0.8 (73)	0.5 (45)

Conclusion: A comprehensive understanding of renal physiology and electrolytes in pregnancy is fundamental for obstetricians and nephrologists, as it enables correct interpretation of laboratory values and identification of deviations from expected gestational adaptations. The profound changes in renal hemodynamics, glomerular filtration rate, electrolyte handling, and osmoregulatory mechanisms during pregnancy require clinicians to recalibrate their diagnostic thresholds recognizing that values considered “normal” in non-pregnant individuals may signify pathology in pregnancy (56–58). Subtle or nonspecific symptoms such as nausea, fatigue, muscle weakness, polyuria, or polydipsia must be interpreted cautiously, as they may represent early manifestations of significant electrolyte disturbances rather than typical physiological complaints of pregnancy.
